# First Principles Calculation of the Effect of Cu Doping on the Mechanical and Thermodynamic Properties of Au-2.0Ni Solder

**DOI:** 10.3390/molecules29174171

**Published:** 2024-09-03

**Authors:** Yan Wei, Hua Dai, Li Chen, Xian Wang, Hongzhong Cai, Jiankang Zhang, Ying Xu, Xingqiang Wang, Junmei Guo, Zhentao Yuan, Xiao Wang

**Affiliations:** 1 Yunnan Precious Metals Laboratory Co., Ltd., Kunming 650106, China; weiyan@ipm.com.cn (Y.W.); daihua@ipm.com.cn (H.D.); chenli@ipm.com.cn (L.C.); wangxian@ipm.com.cn (X.W.); chz@ipm.com.cn (H.C.); zjk@ipm.com.cn (J.Z.); xy88@ipm.com.cn (Y.X.); wxq@ipm.com.cn (X.W.); 2 Kunming Institute of Precious Metals, Kunming 650106, China; 3 Faculty of Materials Science and Engineering, Kunming University of Science and Technology, Kunming 650093, China; wang_xiao@kust.edu.cn

**Keywords:** Au-Ni solder, Cu doping, first principles calculation, mechanical properties, thermodynamic properties

## Abstract

To meet the demands for high-temperature performance and lightweight materials in aerospace engineering, the Au-Ni solder is often utilized for joining dissimilar materials, such as Ti_3_Al-based alloys and Ni-based high-temperature alloys. However, the interaction between Ti and Ni can lead to the formation of brittle phases, like Ti_2_Ni, TiNi, and TiNi_3_, which diminish the mechanical properties of the joint and increase the risk of crack formation during the welding process. Cu doping has been shown to enhance the mechanical properties and high-temperature stability of the Au-Ni brazed joint’s central area. Due to the difficulty in accurately controlling the solid solution content of Cu in the Au-Ni alloy, along with the high cost of Au, traditional experimental trial-and-error methods are insufficient for the development of Au-based solders. In this study, first principles calculations based on density functional theory were employed to analyze the effect of Cu content on the stability of the Au-2.0Ni-xCu (x = 0, 0.25, 0.5, 0.75, 1.0, 1.25 wt%) alloy phase structure. The thermal properties of the alloy were determined using Gibbs software fitting. The results indicate that the Au-2.0Ni-0.25Cu alloy exhibits the highest plastic toughness (B/G = 5.601, ν = 0.416, Cauchy pressure = 73.676 GPa) and a hardness of 1.17 GPa, which is 80% higher than that of Au-2.0Ni. This alloy balances excellent strength and plastic toughness, meeting the mechanical performance requirements of brazed joints. The constant pressure specific heat capacity (C_p_) of the Au-2.0Ni-xCu alloy is higher than that of Au-2.0Ni and increases with Cu content. At 1000 K, the C_p_ of the Au-2.0Ni-0.25Cu alloy is 35.606 J·mol^−1^·K^−1^, which is 5.88% higher than that of Au-2.0Ni. The higher C_p_ contributes to enhanced high-temperature stability. Moreover, the linear expansion coefficient (CTE) of the Au-2.0Ni-0.25Cu alloy at 1000 K is 8.76 × 10^−5^·K^−1^, only 0.68% higher than Au-2.0Ni. The lower CTE helps to reduce the risk of solder damage caused by thermal stress. Therefore, the Au-2.0Ni-0.25Cu alloy is more suitable for brazing applications in high-temperature environments due to its excellent mechanical properties and thermal stability. This study provides a theoretical basis for the performance optimization and engineering application of the Au-2.0Ni-xCu alloy as a gold-based solder.

## 1. Introduction

Ti_3_Al-based alloys have emerged as highly promising materials for lightweight, high-temperature structural applications, primarily due to their high melting points, specific strength, and modulus. These alloys also exhibit excellent high-temperature mechanical properties and superior oxidation resistance, making them ideal for advanced engineering applications [1,2,3]. Notably, Ti_3_Al is the only intermetallic compound extensively used in the aerospace sector, where it has become a preferred material for modern aviation and aerospace engines [4]. One of the key advantages of Ti_3_Al-based alloys is their ability to significantly reduce engine weight compared to traditional high-temperature alloys, thanks to their low density [5]. The integration of Ti_3_Al-based alloys with high-temperature alloys is critical in the manufacturing of engine components. Despite this, most research to date has concentrated on welding Ti_3_Al with titanium alloys [6,7], while studies on welding Ti_3_Al with Ni-based high-temperature alloys remain limited. The combination of Ti_3_Al-based alloys with Ni-based superalloys holds considerable potential, as it leverages their exceptional high-temperature properties while achieving substantial weight reduction.

The joint between Ti_3_Al and nickel-based superalloys, which involves dissimilar materials and operates under prolonged high-temperature conditions, requires not only strong mechanical properties but also high-temperature stability. The selection of an appropriate brazing filler metal is crucial for enhancing the performance of these joints. The gold-based solder is particularly effective due to its excellent thermal conductivity, wettability, oxidation resistance, and high-temperature stability, all while being environmentally friendly. These attributes make it a preferred choice for critical applications, such as satellite connections, aircraft engines, and ceramic components [8,9]. Despite its high cost, the gold-based solder remains indispensable for high-temperature bonding, hermetic sealing, and packaging of dissimilar materials.

For instance, Ren et al. [6] utilized the Au-17.5Ni (wt%) solder to join Ti_3_Al with Ni-based alloys. Their findings indicated that Ni in the solder reacted with the Ti_3_Al matrix, resulting in the formation of brittle phases, such as AlNi_2_Ti and NiTi, which compromised the mechanical properties of the joint. Similarly, Jiang et al. [10] employed a Ti-Zr-Cu-Ni alloy as the brazing filler material for the vacuum brazing TiAl alloy with the Ni-based high-temperature alloy GH536. Their research revealed that compounds, like AlNi_2_Ti and Al_3_NiTi_2_, formed in the brazed joint could act as crack initiation sites under shear stress, leading to a reduction in joint strength. The dissolution enthalpy of Ti in liquid nickel, which is as high as −170 kJ/mol [11], suggests a strong tendency for Ti and Ni to react, forming intermetallic compounds such as Ti_2_Ni, TiNi, and TiNi_3_ [12]. These brittle phases significantly degrade the mechanical properties of the joint, making it prone to cracking during welding. Furthermore, the substantial differences in physical properties, such as the melting point, thermal expansion coefficient, and thermal conductivity, between Ti_3_Al and Ni-based superalloys affect heat conduction during welding, resulting in residual thermal stresses within the weld structure [13,14].

Therefore, controlling the formation of these brittle phases and ensuring a robust metallurgical bond at the interface are critical for achieving a reliable connection between Ti3Al and Ni-based superalloys. Tetsui et al. [15] demonstrated that Au-based solders exhibit strong bonding with TiAl alloys. The addition of 2.0 wt.% Ni to the Au-based solder notably improves ductility, significantly enhances high-temperature creep resistance, and reduces brittleness compared to the conventional 80Au-20Cu solder. In this alloy system, Cu, Ni, and Cr readily form solid solutions without generating intermetallic compounds. Furthermore, Nb and Cu in Ti3Al-based alloys do not form intermetallic compounds. Although Ti, Al, and Cu in Ti_3_Al-based alloys can form intermetallic compounds, these are substantially less brittle and hard than those involving Ni. Notably, Cu’s excellent wettability and plasticity enhance the molten pool’s fluidity and improve the joint’s overall toughness. Chen et al. [16] employed a Ni-Cu alloy as a filler material to achieve dissimilar welding between Ti_3_Al-based alloys and Ni-based high-temperature alloys. Their study revealed that the weld/ Ti3Al interface is characterized by a Ti₂AlNb matrix containing dissolved Ni and Cu, alongside phases such as Al(Cu,Ni)_2_Ti, (Cu,Ni)_2_Ti, and (Nb,Ti) solid solutions. In contrast, the weld/In718 interface primarily consists of (Cu,Ni) solid solutions. The microhardness of the weld was found to be higher than that of the base materials.

These findings indicate that incorporating Cu into the Au-Ni alloy solder could effectively address several challenges, including poor wettability at the Ti_3_Al/Ni interface, the formation of brittle hard phases, and weak bonding strength. This strategy presents a promising avenue for improving the performance of Ti_3_Al-based and Ni-based high-temperature alloys, offering potential advancements in the mechanical properties of brazed joints. However, significant challenges persist, such as the precise control of Cu content in Au-Ni alloys, accurate characterization of their micromechanical properties, and the high cost of Au raw materials. These issues underscore the limitations of conventional “trial and error” approaches in the development of Au-based alloy solders.

To address these challenges, this study utilizes the virtual crystal approximation (VCA) method to construct the Au-2.0Ni-xCu model and employs first principles calculations based on density functional theory (DFT) to examine the influence of Cu content on the phase stability and mechanical properties of Au-2.0Ni-xCu alloys (x = 0, 0.25, 0.50, 0.75, 1.00, 1.25 wt%). Additionally, the thermal properties of these alloys were analyzed using Gibbs software 2 [17,18], elucidating the effect of Cu content on the high-temperature stability of the Au-Ni alloy. The findings of this study aim to provide a theoretical foundation for optimizing the performance and engineering applications of these materials.

## 2. Results

### 2.1. Structure and Stability of the Au-2.0Ni-xCu Alloy

**Figure 1:**
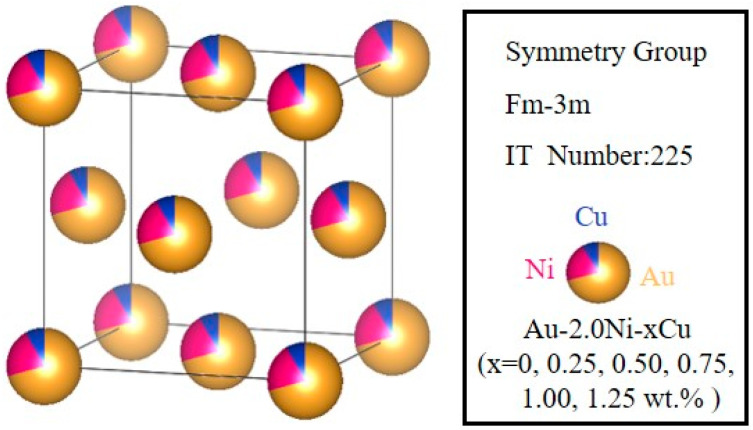
The schematic crystal structure of the Au-2.0Ni-xCu alloy under varying Cu contents.

Given that Cu can dissolve in both Au and Ni to form a solid solution, this study utilizes the virtual crystal approximation (VCA) method to model an Au-2.0Ni-xCu (x = 0, 0.25, 0.50, 0.75, 1.00, 1.25 wt%) solid solution. In this model, Au serves as the matrix, with Ni and Cu acting as solute elements. The virtual crystal approximation (VCA) method is commonly employed to study disordered solid solutions [19]. This approach averages the atomic potentials of two or more elements into a composite potential, offering a simplified representation of substitutional solid solutions [20]. Since the VCA method eliminates the need to construct a supercell, it significantly reduces computational time. This method allows for the seamless integration of Cu and Ni atoms into the Au lattice, thereby providing valuable insights into their interactions within the alloy. The crystal structure of the Au-2.0Ni-xCu alloy is illustrated in Figure 1, with a space group of Fm-3m (space group number 225). To create an initial lattice model for the VCA, this study converted the alloy’s weight percentages to atomic percentages and conducted structural optimization.

To identify the most appropriate exchange–correlation functional, we compared the computational results obtained using the GGA (PBE), GGA (pw91), and LDA (CA-PZ) functionals, as detailed in Table 1. The results indicate that the lattice parameters for Au-2.0Ni-0.25Cu calculated with the LDA (CA-PZ) functional are consistently lower than those derived from the GGA (PBE) and GGA (pw91) functionals. The strong concordance between the GGA (PBE) and GGA (pw91) results suggests that either functional is suitable for this study. Based on this comparison, we selected GGA (PBE) as the exchange–correlation functional for our research. During the structural optimization process, we optimized both atomic positions and unit cell parameters to ensure a stable crystal structure. The optimized lattice constants are presented in Table 2.

To determine the thermodynamic stability of the Au-2.0Ni-xCu alloy, its formation enthalpy (∆H) and cohesive energy ($E_{coh}$) were analyzed. The calculation formula is as follows [21]:

(1) $$∆H=\frac{1}{x+y+z}(E_{tot}-xE_{solid}^{Au}-yE_{solid}^{Ni}-zE_{solid}^{Cu})$$

(2) $$E_{coh}=\frac{1}{x+y+z}(E_{tot}-xE_{atom}^{Au}-yE_{atom}^{Ni}-zE_{atom}^{Cu})$$

where ∆H represents the formation of enthalpy and $E_{coh}$ represents the cohesive energy. The variables x, y, and z denote the number of Au, Ni, and Cu atoms in the unit cell, respectively. $E_{coh}$ is the total energy of the intermetallic compound. $E_{solid}^{Au}$, $E_{solid}^{Ni}$, and $E_{solid}^{Cu}$ are the energies of a single atom in the ground state for Au, Ni, and Cu, respectively. $E_{atom}^{Au}$, $E_{atom}^{Ni}$, and $E_{atom}^{Cu}$ are the energies of isolated atoms of Au, Ni, and Cu, respectively.

**Figure 2:**
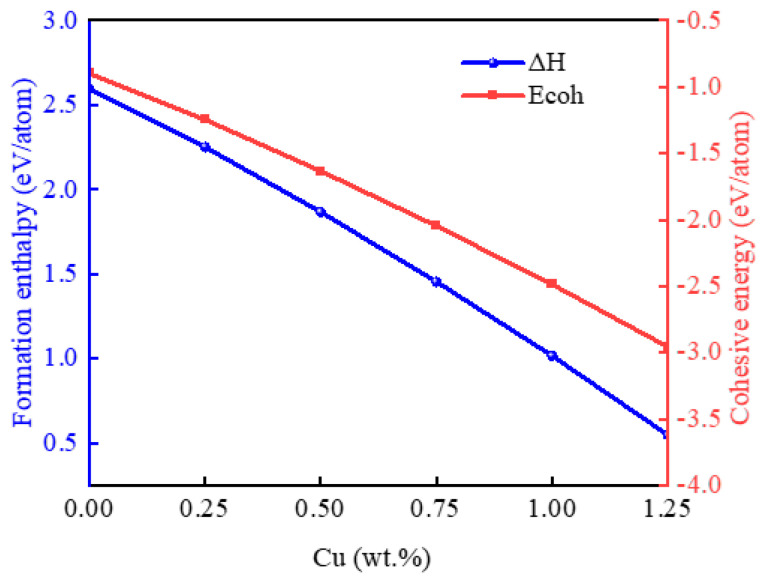
The formation enthalpy and cohesive energy of the Au-2.0Ni-xCu alloy under varying Cu contents.

The calculated values for the enthalpy of formation enthalpy (∆H) and cohesive energy ($E_{coh}$) of the Au-2.0Ni-xCu alloys are presented in Figure 2. A positive enthalpy of formation typically indicates that the reaction is endothermic, requiring heat absorption, while a negative cohesive energy suggests the inherent stability of the compound. As shown in Figure 2, the cohesive energies for all six alloys are negative, confirming their intrinsic stability. Interestingly, the cohesive energy of the Au-2.0Ni-xCu alloys decreases as the Cu content increases, indicating a trend of enhanced stability with higher Cu concentrations. Conversely, the enthalpy of formation for these alloys is positive and increases with the Cu content, suggesting that while the alloy formation is not spontaneous, the difficulty of forming the alloy decreases as the Cu content increases.

### 2.2. Mechanical Properties of the Au-2.0Ni-xCu Alloy

To further determine the mechanical properties of the Au-2.0Ni-xCu alloy, we calculated its elastic constants (C_11_, C_12_, and C_44_) and elastic properties under zero temperature and zero pressure conditions. The results are presented in Table 3. For cubic crystals, mechanical stability is evaluated based on the following criteria: C_11_ > 0, C_44_ > 0, C_11_ > |C_12_|,(C_11_ + 2C_12_) > 0 [22]. The calculated results indicate that the elastic constants of the Au-2.0Ni-xCu alloy meet these criteria, demonstrating that the alloy possesses structural stability under zero temperature and zero pressure conditions.

**Figure 3:**
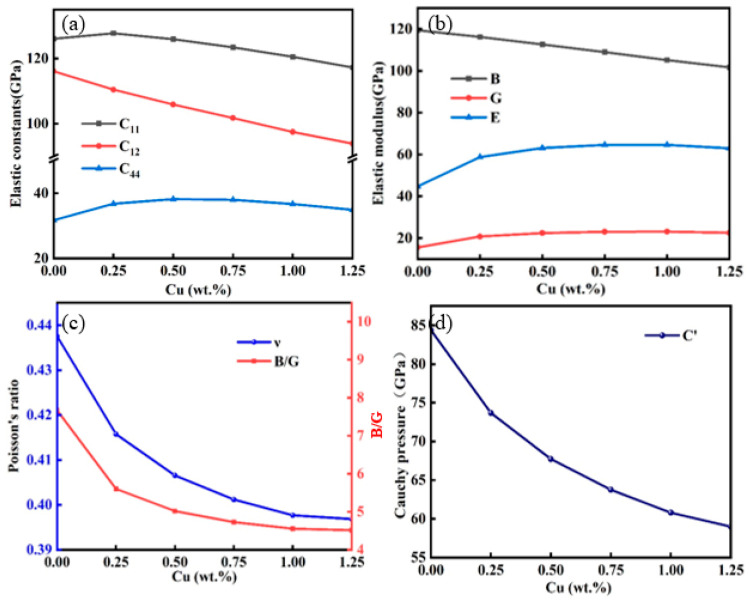
The mechanical constants of the Au-2.0Ni-xCu alloy under varying Cu contents. (**a**) Elastic constants, (**b**) elastic modulus, (**c**) Poisson’s ratio and B/G ratio, (**d**) Cauchy pressure.

Figure 3 illustrates the variations in the bulk modulus, the shear modulus, and Young’s modulus of the Au-2.0Ni-xCu alloy with different Cu contents. The bulk modulus of the alloy decreases as the Cu content increases. Specifically, at a Cu concentration of 1.25 wt%, the bulk modulus drops to 101.676 GPa, which is approximately 14.85% lower than that of pure Au-2.0Ni. Conversely, both the shear modulus and Young’s modulus exhibit an initial increase followed by a decrease with rising Cu content. At 0.25 wt% Cu, the shear modulus and Young’s modulus rise sharply to 20.750 GPa and 58.753 GPa, respectively, marking increases of 33.31% and 31.29% compared to the Au-2.0Ni alloy. Although both moduli continue to increase with additional Cu, a slight reduction is observed when the Cu content reaches 1.25 wt%.

According to Pugh [23], a material is considered ductile if the ratio of the bulk modulus to the shear modulus (B/G) exceeds 1.75; otherwise, it is deemed brittle. Similarly, Poisson’s ratio (ν) provides insight into a material’s ductility; a value greater than 0.26 indicates ductility, while a lower value suggests brittleness. Table 3 shows that the B/G ratio for the Au-2.0Ni-xCu alloy exceeds 1.75, and Poisson’s ratio is above 0.26, confirming the alloy’s plasticity. Additionally, Cauchy pressure is another measure of brittleness and toughness. Positive Cauchy pressure values signify ductility, whereas negative values indicate brittleness. As shown in Figure 3d, the Cauchy pressure values for all Au-2.0Ni-xCu alloys are positive, indicating their toughness; however, this toughness decreases with increasing Cu content. In summary, while the addition of Cu enhances the strength of the Au-2.0Ni-xCu alloy, it also reduces its toughness. This trade-off must be carefully considered in material design.

To analyze the impact of incorporating Cu into the material’s hardness, a semi-empirical formula is employed to assess the hardness of the Au-2.0Ni-xCu alloy. This approach utilizes two commonly applied theoretical models for predicting hardness [24,25], represented by Equations (3) and (4) as follows:

(3) $$H_{\nu 1}=\frac{\left(1-2\nu \right)}{6(1+\nu )}E$$

(4) $$H_{\nu 2}=\frac{\left(1-2\nu \right)}{6(1+\nu )}E$$

where $H_{\nu 1}$ and $H_{\nu 2}$ represent the theoretical hardness of the Au-2.0Ni-xCu alloy, ν denotes Poisson’s ratio, E stands for Young’s modulus, and B and G denote the bulk modulus and shear modulus, respectively.

**Figure 4:**
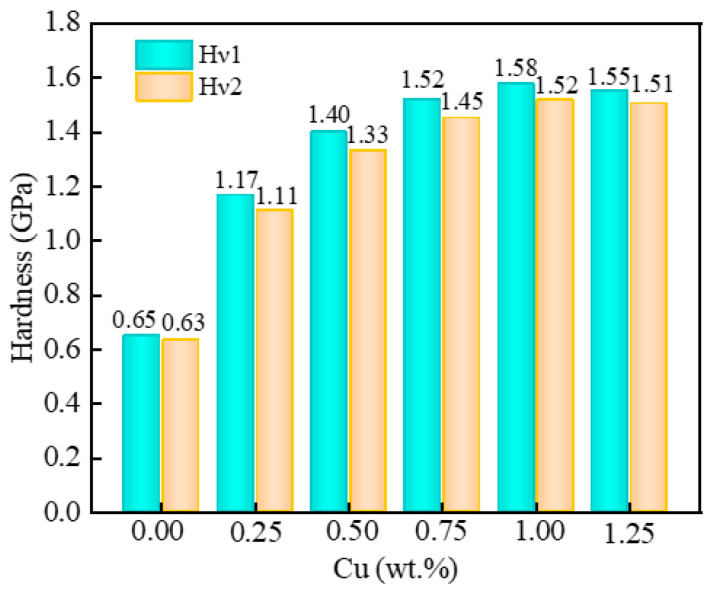
The hardness of the Au-2.0Ni-xCu alloy under varying Cu contents.

The hardness of the Au-2.0Ni-xCu alloy, as calculated using two different models, is illustrated in Figure 4. The figure reveals that, despite minor differences in the hardness values predicted by the two models, their trends are consistent. As the Cu content increases, the hardness of the Au-2.0Ni-xCu alloy first rises and then declines. Specifically, at a Cu content of 0.25 wt%, the alloy shows a hardness of 1.17 GPa, which represents an 80% increase compared to pure Au-2.0Ni. With further increases in Cu content, the hardness continues to improve, reaching a peak value of 1.58 GPa at 1.00 wt% Cu. This peak value indicates a 143% enhancement over Au-2.0Ni. These results demonstrate that the incorporation of Cu significantly enhances the hardness of the Au-2.0Ni-xCu alloy.

**Figure 5:**
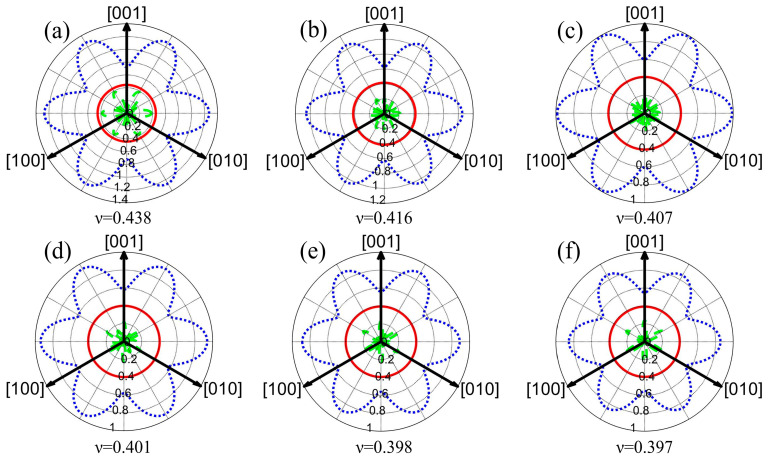
Two-dimensional Poisson’s ratio projection of the Au-2.0Ni-xCu alloy: (**a**) Au-2.0Ni, (**b**) Au-2.0Ni-0.25Cu, (**c**) Au-2.0Ni-0.50Cu, (**d**) Au-2.0Ni-0.75Cu, (**e**) Au-2.0Ni-1.0Cu, (**f**) Au-2.0Ni-1.25Cu (The blue line represents the maximum Poisson’s ratio, the red line represents the average Poisson’s ratio, and the green line represents the minimum Poisson’s ratio).

Figure 5 illustrates the effect of Cu content on Poisson’s ratio of the Au-2.0Ni-xCu alloy, presented as a two-dimensional projection on the (111) plane. The average Poisson’s ratios for the alloys are as follows: Au-2.0Ni (0.438), Au-2.0Ni-0.25Cu (0.416), Au-2.0Ni-0.50Cu (0.407), Au-2.0Ni-0.75Cu (0.401), Au-2.0Ni-1.00Cu (0.398), and Au-2.0Ni-1.25Cu (0.397). All measured values exceed 0.25, indicating ductility, which aligns with the results obtained from the B/G ratio and Cauchy pressure studies. Additionally, Figure 5 shows that as the Cu content increases, the minimum Poisson’s ratio decreases, as represented by a shrinking red circle. This reduction suggests increasing anisotropy in the crystal structure of the Au-2.0Ni-xCu alloys.

**Figure 6:**
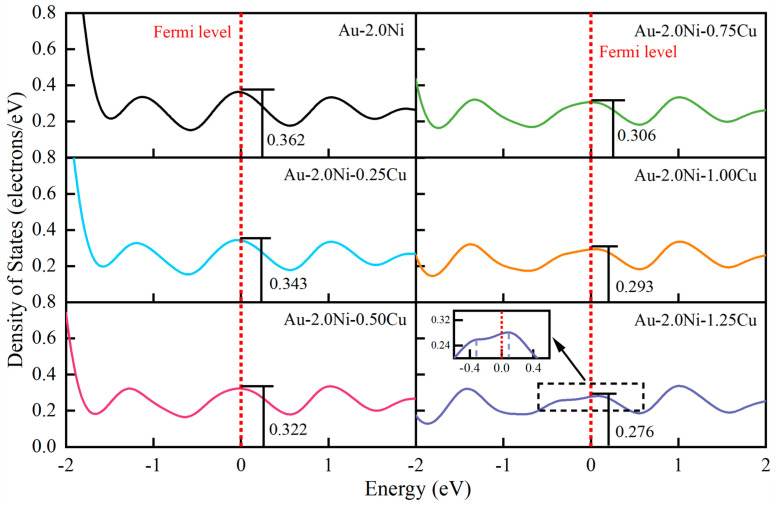
Electronic density of states analysis of the Au-2.0Ni-xCu alloy.

To elucidate the mechanistic effect of Cu doping on the mechanical properties of the Au-2.0Ni alloy, this study analyzed the density of states (DOS) for the Au-2.0Ni-xCu alloy. The results are presented in Figure 6. As shown, the DOS at the Fermi level for Au-2.0Ni-xCu alloys with six different Cu compositions is non-zero, indicating that these alloys maintain metallic properties. With increasing copper (Cu) content, the electron density of states at the Fermi level gradually decreases, which typically correlates with reduced plasticity and enhanced material strength. However, when the Cu content reached 1.25 wt%, the peak in the electronic density of states at the Fermi level exhibited a split. This split suggests a potential decrease in structural stability, an increased likelihood of phase transitions, and consequently, a reduction in material hardness.

The analysis of the mechanical properties of the Au-2.0Ni-xCu alloy indicates that adding an appropriate amount of Cu to the Au-Ni alloy solder can significantly enhance the alloy’s strength, which is crucial for addressing the issues associated with the insufficient strength of the Au-Ni alloy solder, such as premature joint failure. This enhancement contributes to the improved service stability of brazed joints. However, the addition of Cu also results in a reduction in the toughness of the Au-2.0Ni-xCu alloy, which may negatively impact the shear performance of the brazed joint. This trade-off should be carefully considered during the material design process.

### 2.3. Thermal Properties of Au-2.0Ni-xCu Alloys

Understanding the thermodynamic properties of Au-2.0Ni-xCu alloys at varying Cu concentrations is crucial for their application in high-temperature soldering. To evaluate these properties under operational conditions, we used Gibbs software to predict several thermodynamic characteristics over a temperature range from 0 to 1000 K. These characteristics include the constant pressure heat capacity (C_p_), constant volume heat capacity (C_v_), coefficient of thermal expansion (CTE), and other relevant parameters essential for assessing the material’s behavior across different temperature regimes.

**Figure 7:**
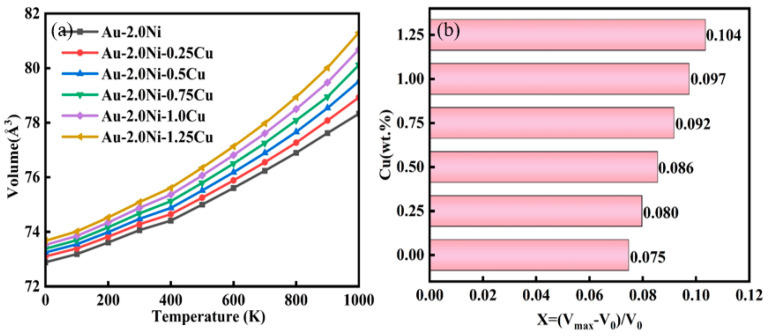
The temperature-volume (T-V) relationship (**a**) and rate of volume change (**b**) of the Au-2.0Ni-xCu alloy under varying Cu contents.

Figure 7 illustrates the temperature-dependent volume change in the Au-2.0Ni-xCu alloy. The data reveal that the volume of the Au-2.0Ni-xCu alloy increases with rising temperature. In the lower temperature range (0–500 K), the volume expansion rate remains relatively stable. However, beyond 500 K, the rate of expansion accelerates significantly. Among the alloys, Au-2.0Ni-1.25Cu exhibits the highest expansion rate. At 1000 K, the volume of Au-2.0Ni-1.25Cu reaches 81.30 Å^3^, which represents a 10.36% increase from its volume at 0 K (73.67 Å^3^) and a 3.83% increase compared to the volume of Au-2.0Ni at 1000 K (78.33 Å^3^).

**Figure 8:**
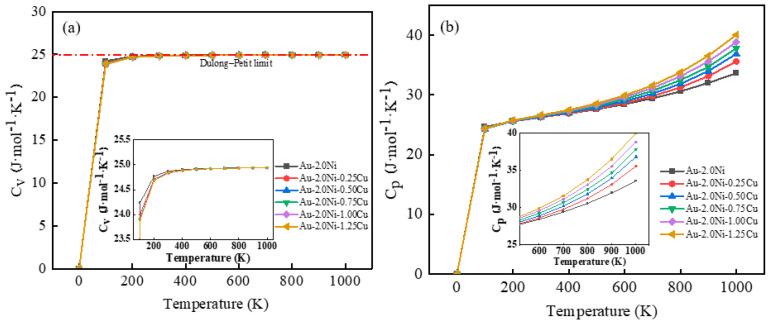
The relationship between the molar heat capacities (C_v_, C_p_) of Au-2.0Ni-xCu alloys at different Cu contents and temperatures: (**a**) C_v_, (**b**) C_p_.

Moreover, Figure 8 illustrates the relationship between the constant volume heat capacity (C_v_) and temperature of the Au-2.0Ni-xCu alloy. It shows that below 100 K, the C_v_ increases rapidly with temperature, with an average slope reaching 0.23. Above 200 K, the rate of increase begins to slow. However, at higher temperatures, the C_v_ decreases with increasing pressure. Beyond 500 K, the C_v_ approaches the limit value predicted by the Dulong–Petit model. The proximity of the material’s constant volume heat capacity to the Dulong–Petit limit at lower temperatures indicates superior thermal stability under high-temperature conditions, minimizing the risk of deformation during service [26].

(5) $$C^{*}=\frac{C}{nR}=\frac{C}{N_{k}}$$

(6) $$C_{v}=3R$$

where the universal gas constant R remains constant at 8.314 J/mol·K and the dimensionless heat capacity C* is fixed at 3. The equation C_V_ = 3R describes a solid with 3N phonons. Given that the total number of atoms in Au-2.0Ni-xCu is four, its specific heat capacity can be calculated using the Dulong–Petit limit to be 24.953 J·mol^−1^·K^−1^.

Figure 8a demonstrates that the constant volume heat capacity (C_v_) of the Au-2.0Ni-xCu alloy stabilizes gradually with increasing temperature. Below 100 K, the C_v_ exhibits rapid growth, which slows significantly above this threshold, resulting in a minimal difference between the C_v_ of Au-2.0Ni-xCu alloy and pure Au. Moreover, it can be seen in Figure 8b that the constant pressure heat capacity (C_p_) of the Au-2.0Ni-xCu alloy follows a similar trend below 100 K and increases sharply as the temperature increases. However, beyond 100 K, the C_p_ behaves distinctly, showing a slower rate of increase. At 1000 K, the maximum C_p_ value of the Au-2.0Ni-1.25Cu alloy reaches 40.07 J·mol^−1^·K^−1^, indicating enhanced heat transfer capabilities that mitigate the risk of mechanical property degradation and potential failure due to heat accumulation at high temperatures.

**Figure 9:**
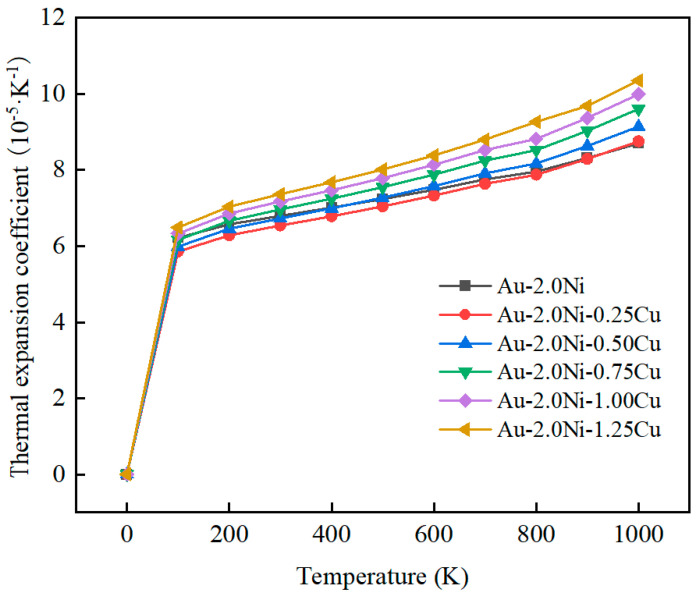
Coefficient of thermal expansion of Au-2.0Ni-xCu alloys.

Meanwhile, the coefficient of thermal expansion (CTE) is a critical parameter indicating how a material responds to temperature changes. The effect of Cu content on the thermal expansion coefficient of the Au-2.0Ni-xCu alloy is shown in Figure 9. As shown in Figure 9, below 100 K, each alloy’s CTE increases significantly with rising temperature, showing an average growth rate of 5.92 × 10^−5^·K^−1^. Beyond 100 K, the rate of CTE growth gradually diminishes. However, beyond 800 K, there is a notable resurgence in the growth rate, particularly evident in the Au-2.0Ni-1.00Cu alloy. At 1000 K, the CTE values for the Au-2.0Ni-0.25Cu, Au-2.0Ni-0.50Cu, Au-2.0Ni-0.75Cu, Au-2.0Ni-1.00Cu, and Au-2.0Ni-1.25Cu alloys are 8.76 × 10^−5^·K^−1^, 9.14 × 10^−5^·K^−1^, 9.60 × 10^−5^·K^−1^, 9.99 × 10^−5^·K^−1^, and 10.35 × 10^−5^·K^−1^, respectively. These represent increases of 0.68%, 5.05%, 10.34%, 14.83%, and 18.97% compared to pure Au-2.0Ni. Higher CTE values can induce thermal stresses in the solder during high-temperature use, potentially leading to joint damage. It is noteworthy that the CTE value of Au-2.0Ni-0.25Cu at 0.25 wt% Cu content aligns closely with that of Au-2.0Ni, suggesting potential benefits in reducing thermal stresses in the Au-2.0Ni-xCu alloy.

Through the above analysis, it is evident that the constant volume heat capacity (C_v_) and constant pressure heat capacity (C_p_) of the Au-2.0Ni-xCu alloy stabilize as the temperature increases. Particularly, at high temperatures, the C_p_ demonstrates excellent heat transfer capabilities, which effectively reduces the risk of failure due to heat accumulation. Additionally, while increasing the Cu content tends to raise the coefficient of thermal expansion (CTE) and may induce thermal stress, it is noteworthy that when the Cu content is 0.25 wt%, the CTE of the Au-2.0Ni-0.25Cu alloy closely aligns with that of pure Au-2.0Ni. This alignment helps to effectively manage thermal stress and enhances the reliability of brazed joints, ensuring superior thermal stress management capabilities.

## 3. Model Construction and Calculation Details

### 3.1. Basic Calculation Parameters

First principles calculations were conducted using the CASTEP code based on density functional theory (DFT) [27]. Electron–ion interactions were modeled using the Vanderbilt ultrasoft pseudopotential scheme, while the exchange–correlation potential was treated within the Perdew–Burke–Ernzerhof (PBE) generalized gradient approximation (GGA) framework [28,29,30]. A plane-wave cutoff energy of 450 eV was employed. Sampling of the first Brillouin zone was achieved using a 4 × 4 × 4 Monkhorst-Pack k-point grid [31]. The equilibrium crystal structure was determined via geometric optimization utilizing the Broyden–Fletcher–Goldfarb–Shanno (BFGS) minimization algorithm [32]. The energy tolerance was set to 2.0 × 10^−5^ eV per atom, with the Hellmann–Feynman forces between ions converged to 0.05 eV/Å, ensuring a maximum ionic displacement of less than 2.0 × 10^−3^ Å. Additionally, finite basis set corrections were implemented to minimize the overall stress tensor to 0.1 GPa. Poisson’s ratio in three-dimensional space was calculated using the ElasticPOST code.

### 3.2. Elastic Constants Analysis

The calculation of material elastic constants, according to the reference, is defined as follows [33]:

(7) $$C_{ijkl}={\left(\frac{\partial \sigma_{ij(x)}}{\partial e_{kl}}\right)}_{X}$$

where $\sigma_{ij}\;$ represents the applied stress, $e_{kl}$, is the strain, X, and χ represent coordinates before and after deformation. For cubic crystals, solving this equation yields three independent elastic constants: C_11_, C_12_, and C_44_. The elastic moduli (bulk modulus, shear modulus, and Poisson’s ratio) related to elasticity can be approximated by the Voigt–Reuss–Hill method [34]. The specific calculation process can be found in Appendix A.

### 3.3. Thermodynamic Properties’ Calculation

This paper employs Gibbs software within the quasi-harmonic Debye approximation model to derive the thermal properties of the Au-2.0Ni-xCu alloy. The calculation process of thermodynamic properties can be found in Appendix A.

## 4. Conclusions

This study employs the virtual crystal approximation method to model the Au-2.0Ni-xCu alloy and applies first principles calculations based on density functional theory to comprehensively investigate its mechanical and thermodynamic properties. The research findings highlight that the Au-2.0Ni-0.25Cu alloy demonstrates superior plastic toughness (B/G = 5.601, ν = 0.416, Cauchy pressure = 73.676 GPa) and a hardness of 1.17 GPa, representing an 80% enhancement compared to Au-2.0Ni. These attributes ensure excellent strength and plastic toughness, meeting the mechanical requirements for brazed joints. Thermodynamic analysis reveals that the constant pressure specific heat capacity (C_p_) of the Au-2.0Ni-xCu alloy exceeds that of Au-2.0Ni and increases with increasing Cu content. At 1000 K, the C_p_ of the Au-2.0Ni-0.25Cu alloy measures 35.606 J·mol^−1^·K^−1^, marking a 5.88% increase over Au-2.0Ni. This elevated C_p_ enhances the alloy’s stability at high temperatures. Moreover, the coefficient of linear expansion (CTE) of the Au-2.0Ni-0.25Cu alloy at 1000 K is 8.76 × 10^−5^·K^−1^, showing only a 0.68% increase compared to Au-2.0Ni. This lower CTE mitigates the risk of solder damage due to thermal stress. Consequently, the Au-2.0Ni-0.25Cu alloy proves highly suitable for brazing applications in high-temperature environments, owing to its outstanding mechanical properties and thermal stability. This study offers theoretical insights into the application of the Au-2.0Ni-xCu alloy as a gold-based solder.

## 5. Patents

This manuscript does not generate any patents.

## Figures and Tables

**Table 1:** The lattice constants of the Au-2.0Ni-0.25Cu alloy.

System	Method	a (Å)	Volume (Å^3^)
Au-2.0Ni-0.25Cu	GGA (PBE)	4.178	72.720
	GGA (pw91)	4.181	73.087
	LDA (CA-PZ)	4.050	66.430

**Table 2:** Equilibrium lattice parameter of the Au-2.0Ni-xCu alloy under varying Cu contents.

Alloys	Atomic Fraction			Lattice Constant/Å (a = b = c)	Angle/° (α = β = γ)
	Au/at%	Ni/at%	Cu/at%		
Au-2.0Ni	93.590	6.410	0	4.175	90
Au-2.0Ni-0.25Cu	92.881	6.379	0.740	4.179	90
Au-2.0Ni-0.5Cu	92.181	6.349	1.470	4.181	90
Au-2.0Ni-0.75Cu	91.500	6.310	2.190	4.184	90
Au-2.0Ni-1.0Cu	90.819	6.281	2.900	4.187	90
Au-2.0Ni-1.25Cu	90.140	6.250	3.610	4.190	90

**Table 3:** Elastic constant (C_ij_), bulk modulus (B), shear modulus (G), Young’s modulus (E), Poisson’s ratio (ν), the B/G ratio and Cauchy pressure of the Au-2.0Ni-xCu alloys prepared with different Cu contents at zero temperature and pressure.

Compounds	C_11_	C_12_	C_44_	B	G	E	ν	B/G	Cauchy Pressure/GPa
	These Units Are All GPa								
Au-2.0Ni	126.067	116.074	31.713	119.405	15.565	44.751	0.438	7.671	84.361
Au-2.0Ni-0.25Cu	127.739	110.448	36.772	116.212	20.750	58.753	0.416	5.601	73.676
Au-2.0Ni-0.50Cu	125.915	105.892	38.176	112.566	22.437	63.117	0.407	5.017	67.715
Au-2.0Ni-0.75Cu	123.427	101.732	37.976	108.964	23.054	64.607	0.401	4.726	63.756
Au-2.0Ni-1.00Cu	120.509	97.462	36.665	105.145	23.093	64.554	0.398	4.553	60.797
Au-2.0Ni-1.25Cu	117.249	93.890	34.910	101.676	22.530	62.940	0.397	4.513	58.981

## Data Availability

No new data were created or analyzed in this study. Data sharing is not applicable to this article.

## Appendix A

1. Elastic constant calculation

For cubic crystals, solving this equation yields three independent elastic constants: C_11_, C_12_, and C_44_. Elastic moduli (bulk modulus, shear modulus, and Poisson’s ratio) related to elasticity can be approximated by the Voigt–Reuss–Hill method [34] as follows:

Voigt average:

(A1) $$B_{V}=\frac{C_{11}+2C_{12}}{3}$$

(A2) $$G_{V}=\frac{C_{11}-C_{12}+3C_{44}}{5}$$

Reuss average:

(A3) $$B_{\;R}=\frac{C_{11}+2C_{12}}{3}$$

(A4) $$G_{R}=\frac{5\left(C_{11}-C_{12}\right)C_{44}}{4C_{44}+3\left(C_{11}-C_{12}\right)}$$

Hill average:

(A5) $$B=B_{H}=\frac{B_{V}+B_{R}}{2}$$

(A6) $$G=G_{H}=\frac{G_{V}+G_{R}}{2}$$

(A7) $$E=\frac{9BG}{3B+G}$$

(A8) $$v=\frac{3B-2G}{6B+2G}$$

Among them, B, G, E, and v are the bulk modulus, the shear modulus, Young’s modulus, and Poisson’s ratio, respectively.

2. Thermodynamic Properties’ Calculation

In this model, the Gibbs free energy in the non-equilibrium state comprises static energy, lattice vibration energy, and energy variations due to volume changes, which are expressed as follows [35]:

(A9) $$G^{*}(x_{opt}(V);p,T)=E(x_{opt}(V))+pV+A_{vib}(x_{opt}\left(V\right);T)$$

In this context, $x_{opt}(V)$ denotes the equilibrium potential energy, intricately dependent on volume and ascertainable through electronic structure calculations. The term pV encompasses enthalpy changes resulting from applied pressure. The vibrational free energy, denoted A_vib_ (V, T), is expressed as follows [36,37,38,39]:

(A10) $$A_{vib}(\Theta ;T)=nkt[\frac{9}{8}\frac{\Theta }{T}+3ln(1-e^{-\frac{\Theta }{T}})-D(\frac{\Theta }{T}))]$$

where D (θ/T) is the Debye integral, which is expressed as follows:

(A11) $$D\left(\Theta /T\right)=\frac{3}{{\left(\Theta /T\right)}^{3}}\int_{0}^{\Theta /T}\frac{x^{3}}{e^{x}-1}dx$$

In the formula, Θ represents the Debye temperature, which can be calculated by the following formula [36]:

(A12) $$\Theta =\frac{\hbar }{k}{\left[6\pi^{2}V^{1/2}n\right]}^{1/3}f(\sigma )\sqrt{\frac{B_{s}}{M}}$$

In the given equation, the symbol n denotes the number of atoms constituting the molecule. Additionally, the variables σ, M, and B_s_ signify Poisson’s ratio, molar mass, and the adiabatic bulk modulus, respectively.

B_s_ can be calculated by the following formula [17]:

(A13) $$B_{S}=-{V\left(\frac{\partial p}{\partial V}\right)}_{S}={V\left(\frac{\partial^{2}U}{\partial V^{2}}\right)}_{S}=B_{T}\left(1+\gamma th\alpha T\right)$$

The function f(σ) is explicitly delineated as follows [36,37]:

(A14) $$(\sigma )={\left\{{\left[3\left[2(\frac{2}{3}\cdot \frac{1+\sigma }{1-2\sigma }{)}^{\frac{3}{2}}+{\left(\frac{1}{3}\cdot \frac{1+\sigma }{1-\sigma }\right)}^{\frac{3}{2}}\right]\right]}^{-1}\right\}}^{\frac{1}{3}}$$

Θ is thus a function of **x** through its dependence on *V*, *B_S_*_,_ and *σ*, and the latter can be considered as a constant for each solid to a good approximation.

We are now in a position to obtain the (*p*, *T*) equilibrium situation by minimizing

(A15) $$G^{*}\left(V;p,T\right)=E\left(V\right)+pV+A_{Vvib}\left(\Theta \left(V\right);T\right)$$

with respect to *V* or, equivalently, by solving

(A16) $${\left(\frac{{\partial G}^{*}(V;p,T)}{\partial V}\right)}_{p,T}=0$$

This process generates an equilibrium volume, denoted *V*_opt_(*p*,*T*), at a given pressure *p* and temperature *T*. Utilizing this equilibrium volume, we can derive the isothermal bulk modulus within the framework of equilibrium thermodynamic relations as follows [36]:

(A17) $$B_{T}\left(p,T\right)=-{\left(V\frac{\partial p}{\partial V}\right)}_{T}=V_{opt}({\frac{\partial^{2}G^{*}\left(V;p,T\right)}{\partial V^{2}})}_{p,T}\left(V_{opt}\right)$$

by virtue of the condition (23) that defines the equilibrium volume.

Upon reaching equilibrium, the molar heat capacities at constant volume (C_V_) and constant pressure (C_p_), as well as the coefficient of thermal expansion (α) of the crystal, can be determined using the following equations [18]:

(A18) $$C_{v}=3\mathrm{nk}\left[4D\left(\frac{\Theta }{T}\right)-\;(\frac{3\Theta /T}{\Theta /T-1})\right]$$

(A19) $$C_{p}=C_{v}(1+\gamma \alpha T)$$

(A20) $$\alpha =\gamma C_{v}/B_{T}V$$
